# Efficacy and safety of immune checkpoint inhibitors in solid tumor patients combined with chronic coronary syndromes or its risk factor: a nationwide multicenter cohort study

**DOI:** 10.1007/s00262-024-03747-w

**Published:** 2024-06-08

**Authors:** Chao Liu, Yuli Ruan, Rui Huang, Lin Fang, Tong Wu, Ying Lv, Luying Cui, Yuanyu Liao, Bojun Wang, Zhuo Chen, Dan Su, Yue Ma, Shuling Han, Xin Guan, Jie Cui, Yang Yao, Yao Wang, Mengmeng Wang, Ruiqi Liu, Yanqiao Zhang

**Affiliations:** 1 Department of Gastrointestinal Medical Oncology, Harbin Medical University Cancer Hospital, Harbin, China; 2 Clinical Research Center for Colorectal Cancer in Heilongjiang, Harbin, China; 3 Key Laboratory of Tumor Immunology in Heilongjiang, Harbin, China; 4 Cancer Diagnosis and Treatment Center, Shangluo Central Hospital, Shangluo, China; 5 Phase I Clinical Research Center, The Affiliated Hospital of Qingdao University, Qingdao, China; 6 Department of Cardiology, The Second Affiliated Hospital of Harbin Medical University, Harbin, China; 7 Department of Oncology, Daqing Oilfield General Hospital, Daqing, China; 8 Department of Oncology Medicine, Central People’s Hospital of Zhanjiang, Zhanjiang, China; 9 Pulmonary and Critical Care Medicine Unit 2, The Second Affiliated Hospital of Harbin Medical University, Harbin, China; 10 The Second Department of Oncology, Beidahuang Industry Group General Hospital, Harbin, China; 11 Department of Radiation Oncology, Sun Yat-Sen University Cancer Center, Guangzhou, China

**Keywords:** Immune checkpoint inhibitors, Chronic coronary syndromes, Malignant solid tumor, Prognosis, Immune-related adverse events

## Abstract

**Background:**

Although, immune checkpoint inhibitors (ICIs) have been widely applied in the therapy of malignant tumors, the efficacy and safety of ICIs in patients with tumors and pre-existing CAD, especially chronic coronary syndromes (CCS) or their risk factors (CRF), is not well identified.

**Methods:**

This was a nationwide multicenter observational study that enrolled participants who diagnosed with solid tumors and received ICIs therapy. The main efficacy indicators were progression-free survival (PFS) and overall survival (OS), followed by objective response rate (ORR) and disease control rate (DCR). Safety was assessed by describing treatment-related adverse events (TRAEs) during ICIs therapy evaluated by the Common Terminology Criteria for Adverse Events 5.0 (CTCAE 5.0).

**Results:**

In the current research, we retrospectively analyzed the data of 551 patients diagnosed with solid tumors and received ICIs therapy, and these patients were divided into CCS/CRF group and non-CCS/CRF group. Patients with CCS/CRF had more favorable PFS and OS than patients without CCS/CRF (*P* < 0.001) and the pre-existing CCS/CRF was a protective factor for survival. The ORR (51.8% vs. 39.1%) and DCR (95.8% vs. 89.2%) were higher in CCS/CRF group than in non-CCS/CRF group (*P* = 0.003, *P* = 0.006). In this study, there was no significant difference in treatment-related adverse events (TRAEs), including immune-related adverse events (irAEs), between the two groups.

**Conclusions:**

We concluded that ICIs appear to have better efficacy in malignant solid tumor patients with pre-existing CCS/CRF and are not accompanied by more serious irAEs.

**Supplementary Information:**

The online version contains supplementary material available at 10.1007/s00262-024-03747-w.

## Introduction

Cancer and coronary artery disease (CAD) are two of the most common causes of death, and they frequently coexist, especially as the world’s population ages [1]. CAD can develop prior to or following cancer diagnosis, as well as arise a side effect of cancer treatment [2]. The risk factors of CAD include diabetes mellitus, hypertension, smoking, hyperlipidemia, obesity, homocystinuria, and psychosocial stress. A study found that 18.0% of cancer patients were accompanied by CAD or its risk factor (CRF), the most common type was hypertension (10.8%), followed by diabetes mellitus (5.3%) and hyperlipidemia (1.2%) [3]. Certain chemotherapeutic agents and ionizing radiation are well-established to cause accelerated coronary atherosclerosis and CAD [2, 4]. Immune checkpoint inhibitors (ICIs) have revolutionized treatment for various cancer and significantly improved survival outcomes for patients with immunogenic tumors, such as melanoma, hepatocellular carcinoma (HCC), non-small cell lung cancer (NSCLC), and MSI-H colorectal cancer (CRC) [5–7]. The increased T-cell activation from ICIs promotes enhanced anti-tumor activity and induces immune-related adverse events (irAEs) [8]. These unique treatment-specific toxicities commonly affect the skin, gut, endocrine glands, liver, lungs, and myocardium [8–12].

Incidence of cardiovascular irAEs (CV-irAEs) are rare but fatal. The most common CV-irAE is myocarditis, although pericarditis, arrhythmia, and ventricular dysfunction have also occurred in patients treated with ICIs [11, 13, 14]. An observational study from VigiBase showed that more than 80% of CV-irAEs cases were severe, with mortality rates reaching 50% (61/122) of myocarditis cases and 21% (20/95) of pericardial disease cases [15]. Moreover, CV-irAEs were more common with combination ICIs therapy compared to monotherapy [15, 16]. To date, for tumor patients with CAD or CRF, few studies have investigated the association between CAD/CRF and the application of ICIs in these patients. Therefore, it brings great confusion to the physicians about the safety and efficacy of ICIs in cancer patients accompanied by CAD/CRF.

In this real-world study, we conducted a retrospective analysis that included 216 patients treated with ICIs from 8 clinical centers across China with pre-existing CAD/CRF at the time of their diagnosis of advanced solid tumors to establish the safety and efficacy of ICIs in this population. Following the 2019 European Society of Cardiology guidelines on CAD, we only collected chronic coronary syndromes (CCS) patients and excluded patients with acute coronary syndrome (ACS) in this study. Obviously, these patients will not receive any anti-tumor therapy until their heart disease stabilizes. Meanwhile, we collected patients with concomitant risk factors of hypertension, diabetes, and dyslipidemia, while excluding those with smoking, obesity, and genetic factors. This study will provide preliminary support for further assessment of the ICIs regimen for solid tumor patients with pre-existing CCS/CRF.

## Methods

### Patients

**Fig. 1:**
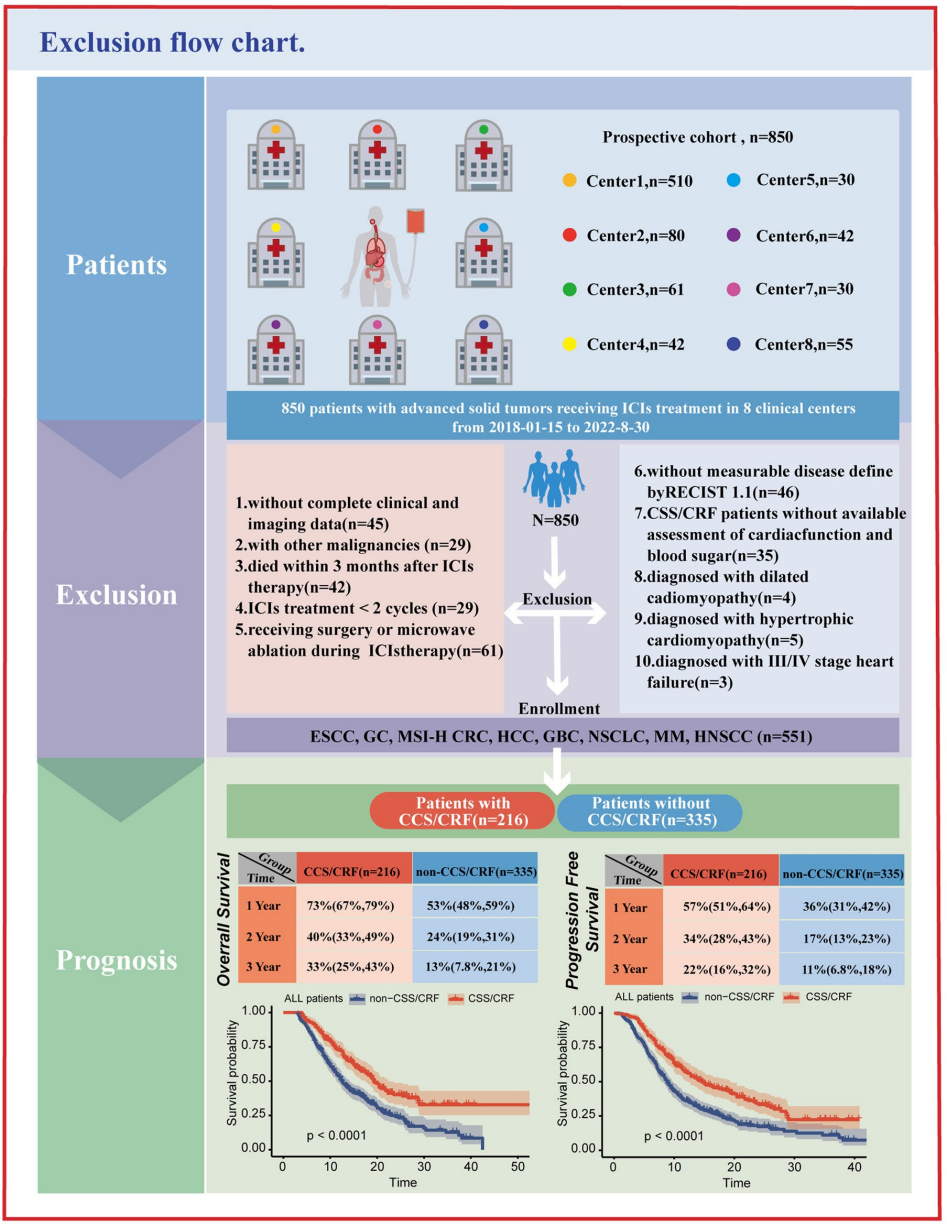
Exclusion flow chart. ICIs: immune checkpoint inhibitors, RECIST 1.1: Response Evaluation Criteria in Solid Tumors 1.1, ESCC: esophageal squamous cell carcinoma, GC: gastric cancer, MSI: microsatellite instability, CRC: colorectal cancer, HCC: hepatocellular carcinoma, BTC: biliary tract carcinoma, NSCLC: non-small cell lung cancer, MM: melanoma, HNSCC: squamous cell carcinoma of head and neck, CCS: chronic coronary syndromes, CRF: coronary risk factors

We retrospectively reviewed patients with malignant solid tumors and pre-existing CCS/CRF who received ICIs therapies from nationwide 8 clinical centers, including Harbin Medical University Cancer Hospital (Harbin, China), Shangluo Central Hospital, (Shangluo, China), The Affiliated Hospital of Qingdao University (Qingdao, China), The Second Affiliated Hospital of Harbin Medical University (Harbin, China), Daqing Oilfield General Hospital, (Daqing, China), Central People’s Hospital of Zhanjiang (Zhanjiang, China), Beidahuang Industry Group General Hospital (Harbin, China), and Sun Yat-sen University Cancer Center (Guangzhou, China), between 2018-01-15 and 2022-08-30. The sample size was determined based on the number of cases in these areas during the study period. These tumors included esophageal squamous cell carcinoma (ESCC), gastric cancer (GC), mismatch repair protein-deficiency/microsatellite instability-high colorectal cancer (dMMR/MSI-H CRC), hepatocellular carcinoma (HCC), biliary tract carcinoma (BTC), non-small cell lung cancer (NSCLC), melanoma, and squamous cell carcinoma of head and neck (HNSCC). The inclusion criteria were as follows: (1) aged > 18 years; (2) confirmed diagnosis of malignant solid tumors; (3) received ICIs therapies (anti-CTLA-4, anti-PD-1, or anti-PD-L1 antibodies) and treatment cycle > 2; (4) with complete clinicopathologic and imaging data; (5) with pre-existing CCS/CRF. Patients not suitable for ICIs therapy according to the National Comprehensive Cancer Network (NCCN) Guidelines, those with combined other malignant tumors, received surgery or radiotherapy during ICIs therapy, and died within 3 months after ICIs therapy were excluded. All patients underwent medical examination, including medical history, physical, hematological, coronary vascular imaging (coronary CT scan or selective coronary angiography) before enrollment in this study within six months. The Non-CCS/CRF group was scrupulously selected by excluding individuals with coronary artery disease and its risk factor, including arterial hypertension, hyperlipidemia, and diabetes. Finally, 299 patients were excluded, and 551 patients were assigned to either a CCS/CRF group (n = 216) or a non-CCS/CRF group (n = 335) (Fig. 1). Furthermore, we enrolled 125 patients with stage I-III dMMR/MSI-H CRC who underwent a primary resection at the HMUCH between 2013 and 01-04 and 2015-12-30 to explore the difference of infiltrating immune cells in pathological tissues between CCS/CRF group (N = 44) and non-CCS/CRF (N = 81) group (sTable 1).This study complied with the principles of the Declaration of Helsinki and was approved by the ethics committee of the Harbin Medical University Cancer Hospital (retrospective study approval KY2021-037). This study was approved by all institutional review boards, and all participants provided written informed consent.

### Definition of CCS and CCS-associated risk factors (CRF)

For the definition of chronic coronary syndromes (CCS) and CCS-associated risk factors (CRF), including hypertension, diabetes mellitus and dyslipidemia, we refer to the 2019 European Society of Cardiology (ESC) guidelines [17–20]. The most frequently encountered clinical scenarios in patients with suspected or established CCS are: (i) patients with suspected CAD and ‘stable’ anginal symptoms, and/or dyspnoea; (ii) patients with new onset of heart failure (HF) or left ventricular (LV) dysfunction and suspected CAD; (iii) asymptomatic and symptomatic patients with stabilized symptoms < 1 year after an ACS, or patients with recent revascularization; (iv) asymptomatic and symptomatic patients > 1 year after initial diagnosis or revascularization; (v) patients with angina and suspected vasospastic or microvascular disease; and (vi) asymptomatic subjects in whom CAD is detected at screening. Hypertension is defined as office SBP values at least 140 mmHg and/or diastolic BP (DBP) values at least 90 mmHg. Diagnosis of diabetes mellitus (DM) is based on HbA1c ≥ 6.5% (48 mmol/mol) or FPG ≥ 7.0 mmol/L (126 mg/dL), and on OGTT if still in doubt. The criteria of dyslipidemia are: total cholesterol ≥ 6.2 mmol/L, low density lipoprotein cholesterol ≥ 4.1 mmol/L, triglyceride ≥ 2.3 mmol/L, high density lipoprotein cholesterol < 1.0 mmol/L, or more than one of the above four items.

### Efficacy and safety assessments

Efficacy was evaluated with treatment response according to Response Evaluation Criteria in Solid Tumors 1.1 (RECIST 1.1) [21] and progression-free survival (PFS) is defined as the time from the initiation of ICIs therapy to tumor progression or death. Overall survival (OS) was defined as the time from the first medication date to the last follow-up date or death. The objective response rate (ORR) was categorized as complete response (CR) and partial response (PR). Disease control rate (DCR) was categorized as CR, PR, and stable disease (SD), excluding progressive diseases (PD). The evaluation of ICIs therapy's efficacy was based on imaging and conducted every 6–12 weeks.

Safety was assessed by describing treatment-related adverse events (TRAEs) during ICIs therapy evaluated by the Common Terminology Criteria for Adverse Events 5.0 (CTCAE 5.0) [22]. We also referred to American Society of Clinical Oncology (ASCO) Clinical Practice Guideline regarding irAEs for a more comprehensive description of CV-irAEs [14]. The basic assessment indexes for CV-irAEs include electrocardiography (ECG), troponin, brain natriuretic peptide (BNP), and echocardiogram.

### Immunochemistry (IHC)

Primary tumor paraffin sections of 4 μm were processed for immunochemistry (IHC) to evaluate CD8 + cells according to the following protocol: roast, deparaffinized, and rehydration before performing heat-mediated antigen retrieval with EDTA buffer (C1034; Solarbio, China) pH 9.0, inactivation endogenous peroxidase activity with 3% H2O2 (PV-6001; Nakasugi Jinqiao, Beijing, China), incubation with antibody against CD8 (ab101500, 1:500; Abcam, Cambridge, UK) at 4 °C overnight, exposure to DAB IHC Detection Kit (ZLI-9019; Nakasugi Jinqiao, Beijing, China) after incubation with the goat anti-rabbit secondary antibodies (PV-6001; Nakasugi Jinqiao, Beijing, China), and counterstained with Mayer’s Hematoxylin solution.

### Statistical analysis

Clinicopathologic features of patients in the CCS/CRF group and the non-CCS/CRF group were compared by Mann–Whitney U test, χ2 test, T-test, or Fisher's exact test. Kaplan–Meier survival analysis and log-rank tests were used to compare the differences in PFS between different cardiovascular functions. Univariate and multivariate analyses were evaluated by the Cox regression model to determine the risk factors associated with PFS. All statistical tests were two-sided, and *P* < 0.05 was considered statistically significant. Statistical analyses were performed using the R statistical language (R version 4.2.2).

## Results

### Patients and disease characteristics

Of 551 patients enrolled in this study, a total of 161 (29.2%) were diagnosed with ESCC, 137 (24.9%) were GC, 22 (4.0%) were MSI-H CRC, 61 (11.1%) were HCC, 53 (9.6%) were BTC, 110 (20.0%) were NSCLC, 2 (0.4%) were melanoma, and 5 (0.9%) were HNSCC. It was found that 367 (66.6%) patients received ICIs therapy combined with chemotherapy, other treatment regimens consisted of ICIs monotherapy (N = 61, 11.1%), combined with targeted therapy (N = 66, 12.0%), combined with chemotherapy and targeted therapy (N = 57, 10.3%). There were 120 (21.8%) patients with a history of radical treatment and 419 (76.0%) patients who were in the stage of first-line ICIs therapy. The detailed information was listed in Table 1, and statistically intergroup differences were observed in patients' age and body mass index (BMI). Types of pre-existing CCS/CRF included chronic coronary syndrome (N = 28, 12.96%), hypertension (N = 93, 43.06%) and diabetes mellitus (N = 47, 21.76%), dyslipidemia (N = 87, 40.28%). Additionally, there were 33 cases with a combination of two conditions and 3 cases with a combination of all three (Supplementary Table 2).

**Table 1:** Clinicopathologic factors of 551 patients with malignant solid tumors patients from 8 nationwide hospitals

Clinicopathological features	Non_CCS /CRF	CCS/CRF (N = 216)	*P* value
	(N = 335)		
Age (yr)			0.001
Mean (IQR)	60 (54, 66)	63 (56, 69)	
Range	(21–81)	(34–85)	
Sex, N (%)			0.439
Female	76 (22.7)	43 (19.9)	
Male	259 (77.3)	173 (80.1)	
BMI (kg/m^2)			0.006
Mean (IQR)	22.0 (20.1, 24.2)	22.9 (20.6, 25.3)	
Range	(14.19–34.38)	(14.84–31.89)	
Cancer type, N (%)			0.697
ESCC	92 (27.5)	69 (31.9)	
GC	87 (26.0)	50 (23.1)	
MSI-H CRC	13 (3.88)	9 (4.17)	
HCC	33 (9.85)	28 (13.0)	
BTC	36 (10.7)	17 (7.87)	
NSCLC	69 (20.6)	41 (19.0)	
MM	1 (0.30)	1 (0.46)	
HNSCC	4 (1.19)	1 (0.46)	
Primary tumor resection, N (%)			0.520
No	259 (77.3)	72 (79.6)	
Yes	76 (22.7)	44 (20.4)	
Treatment line, N (%)			0.104
First line	245 (73.1)	174 (80.6)	
Second line	65 (19.4)	33 (15.3)	
Third line	25 (7.46)	9 (4.17)	
ICIs class, N (%)			0.628
ICIs monotherapy	37 (11.0)	24 (11.1)	
With chemotherapy	218 (65.1)	149 (69.0)	
With targeted therapy	41 (12.2)	25 (11.6)	
With chemotherapy and targeted therapy	39 (11.6)	18 (8.33)	

*CCS* chronic coronary syndromes *CRF* coronary risk factors *IQR* interquartile range *BMI* body mass index *ESCC* esophageal squamous cell carcinoma *GC* gastric cancer, MSI: microsatellite instability *CRC* colorectal cancer *HCC* hepatocellular carcinoma *BTC* biliary tract carcinoma *NSCLC* non-small cell lung cancer *MM* melanoma *HNSCC* squamous cell carcinoma of head and neck, ICIs: immune checkpoint inhibitors

### Patients with CCS/CRF benefit from the better short-term effect from ICIs therapy

**Fig. 2:**
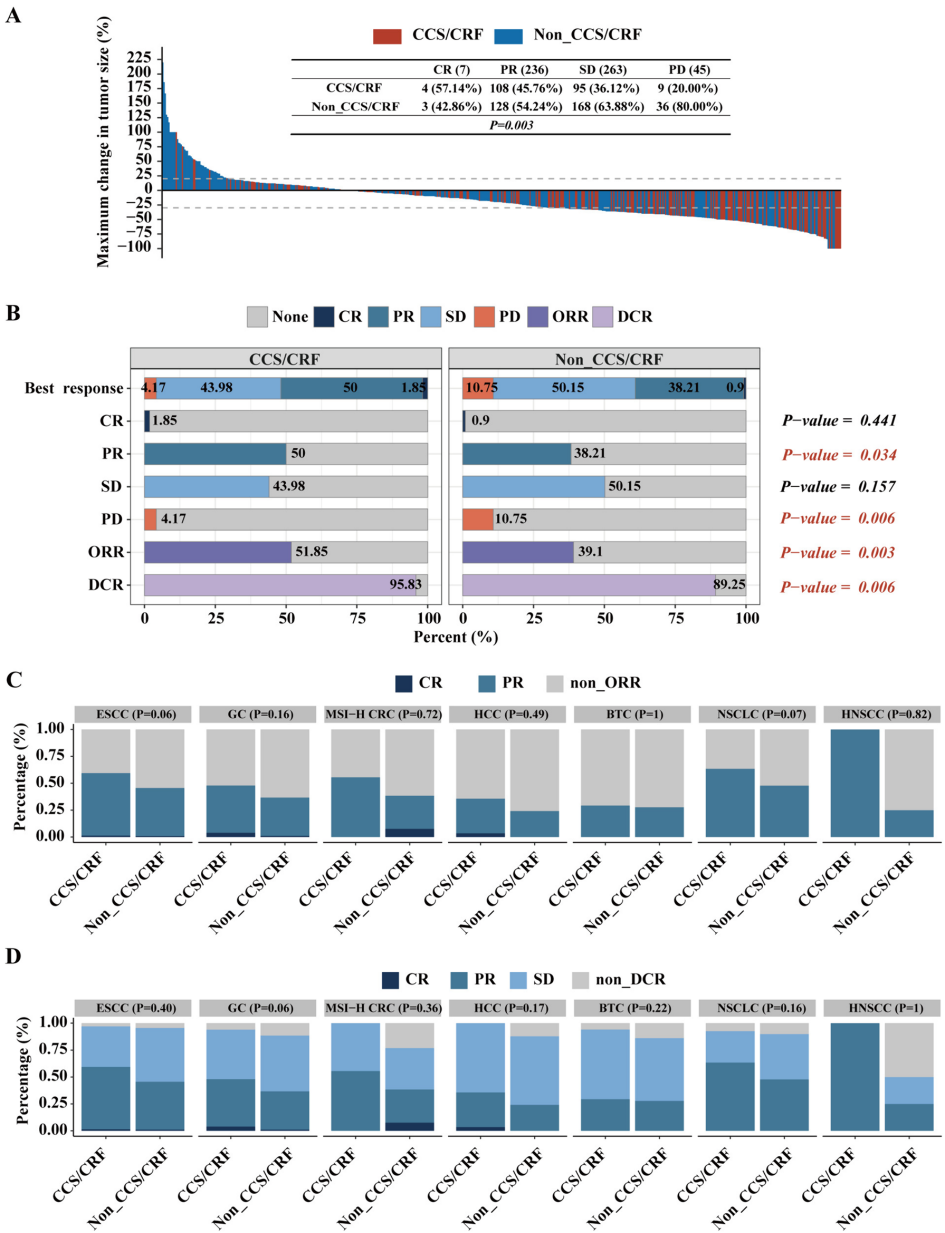
Best of response of ICIs in tumor patients from 8 nationwide hospitals. **A** Best percentage change in target lesion size from baseline of 551 enrolled malignant solid tumor patients. **B** Evaluation of the curative effect of the different groups. **C** A stacked bar chart illustrating the proportions of ORR for various tumor types between different groups. **D** A stacked bar chart illustrating the proportions of DCR for various tumor types between different groups. CR: complete response, PR: partial response, SD: stable disease, PD: progressive disease, ORR: overall response rate, DCR: disease control rate, ESCC: esophageal squamous cell carcinoma, GC: gastric cancer, MSI: microsatellite instability, CRC: colorectal cancer, HCC: hepatocellular carcinoma, BTC: biliary tract carcinoma, NSCLC: non-small cell lung cancer, HNSCC: squamous cell carcinoma of head and neck, CCS: chronic coronary syndromes, CRF: coronary risk factors

The changes of tumor size in 551 patients at the best of response (BOR) for ICIs therapy are shown in Fig. 2A. The BOR in CCS/CRF group and non-CCS/CRF group patients were shown in Fig. 2B. The ORR and DCR in the 551 patients were 44.1% (243/551) and 91.8% (506/551), respectively. In CCS/CRF group, 4 patients achieved CR and 108 patients showed PR, while in the non-CCS/CRF group, we observed PR in 128 patients, and CR in 3 patients. The ORR was higher in CCS/CRF group (112/216, 51.85%) than in the non-CCS/CRF group (131/335, 39.1%) (*P* = *0.003*). Furthermore, the DCR was higher in CCS/CRF group (207/216, 95.8%) than in the non-CCS/CRF group (299/335, 89.3%) (*P* = *0.006*). In the best response of CCS/CRF group and non-CCS/CRF group, PR (50% vs. 38.2%, *P* = *0.034*) and PD (4.2% vs. 10.8%, *P* = *0.006*) showed a statistical difference. In subgroups ESCC, GC, MSI-H CRC, HCC, BTC, NSCLC and HNSCC, CCS/CRF group patients showed better therapeutic effects in both ORR and DCR, although it did not reach statistical significance (all *P* > 0.05). (Fig. 2C and D).

### Patients with CCS/CRF benefit from better long-term effect from ICIs therapy

**Fig. 3:**
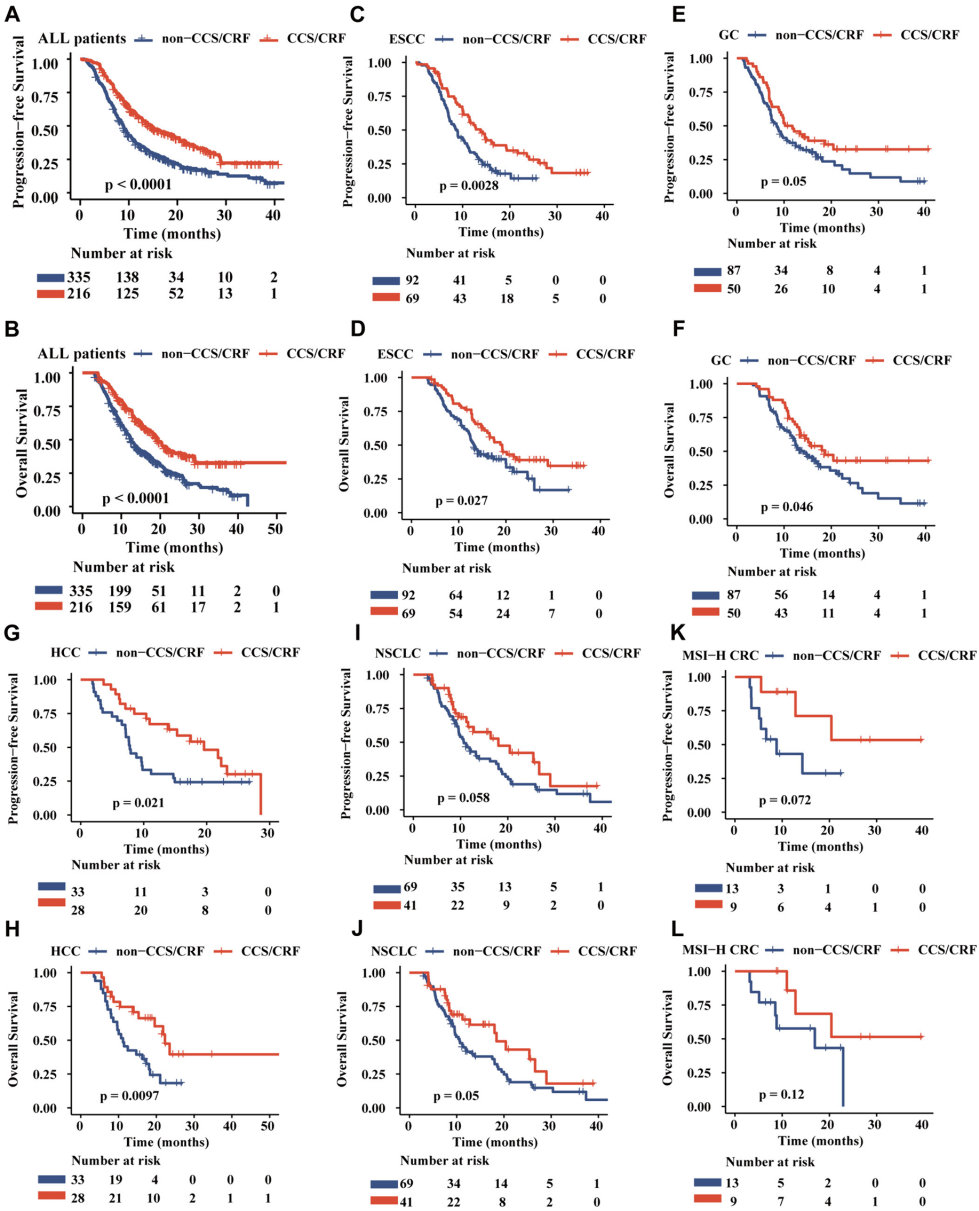
Survival analysis of tumor patients from 8 nationwide hospitals receiving immune checkpoint inhibitors treatments. Kaplan–Meier curves of PFS for **A** all malignant solid tumor patients, **C** ESCC, **E** GC, **G** HCC, **I** NSCLC, **K** MSI-H CRC. Kaplan–Meier curves of OS for **B** all malignant solid tumor patients, **D** ESCC, **F** GC, **H** HCC, **J** NSCLC, **L** MSI-H CRC. PFS: Progression-free survival, OS: Overall survival, ESCC: esophageal squamous cell carcinoma, GC: gastric cancer, MSI: microsatellite instability, CRC: colorectal cancer, HCC: hepatocellular carcinoma, NSCLC: non-small cell lung cancer, HNSCC: squamous cell carcinoma of head and neck, CCS: chronic coronary syndromes, CRF: coronary risk factors

Patients with CCS/CRF showed better long-term efficacy after receiving ICIS, including PFS and OS. By the last follow-up date (2023-08-30), 393 patients (71.3%) had experienced progressive disease, and 344 patients (62.4%) died. The progression and death of patients in the CCS/CRF group and those in the non-CCS/CRF group were respectively observed in 132 vs. 261 patients (61.1% vs. 77.9%) and 72 vs. 272 patients (33.3% vs. 81.2%). The Kaplan–Meier curve showed that patients with CCS/CRF had significantly longer survival for PFS and OS (*P* < 0.001) (Fig. 3A, B). In patients with ESCC, GC, HCC and NSCLC, the CCS/CRF group has longer PFS than non-CCS/CRF group (Fig. 3C, E, G and I) and have a longer survival for OS in the CCS/CRF group than non-CCS/CRF (Fig. 3D, F, H and J). A similar trend was shown in MSI-H CRC and BTC patients, although not statistically significant (Fig. 3K, L and Supplementary Fig. 1A, B). Multivariate analysis demonstrated that pre-existing CCS/CRF (HR: 0.58, 95% CI 0.46–0.71, *P* < 0.001) independently influenced PFS (Table 2). Additionally, pre-existing CCS/CRF (HR: 0.57, 95% CI 0.45–0.72, *P* < 0.001) were identified as independent prognostic factors for OS (Table 3).

**Table 2:** Univariable and multivariable analysis of progression-free survival

	Progression-free survival					
	Univariable analyses			Multivariable analyses		
	HR	95% CI	*P* value	HR	95% CI	*P* value
CCS/CRF						
Yes versus No	0.58	0.47–0.71	< 0.001	0.58	0.46–0.71	< 0.001
Age (yr)	0.99	0.98–1.00	0.181			
Sex	1.01	0.79–1.28	0.949			
Male versus Female						
BMI						
18.5–23.9 vs < 18.5	0.87	0.63–1.19	0.379	0.83	0.60–1.15	0.251
> = 24 vs < 18.5	0.68	0.48–0.97	0.031	0.73	0.51–1.04	0.086
Primary tumor resection						
Yes versus No	1.06	0.83–1.34	0.640			
Treatment line						
Second line versus First line	1.10	0.85–1.42	0.482	1.09	0.83–1.42	0.536
Third line versus First line	1.64	1.12–2.41	0.011	1.53	1.03–2.27	0.036
ICIs class						
With chemotherapy versus ICIs monotherapy	1.30	0.92–1.84	0.136	1.41	0.99–2.02	0.059
With targeted therapy versus ICIs monotherapy	1.59	1.03–2.45	0.036	1.62	1.04–2.53	0.033
With chemotherapy and targeted therapy versus ICIs monotherapy	1.16	0.75–1.81	0.505	1.21	0.77–1.89	0.407

*CCS* chronic coronary syndromes *CRF* coronary risk factors *BMI* body mass index *ICIs* immune checkpoint inhibitors

**Table 3:** Univariable and multivariable analysis of overall survival

	Overall survival					
	Univariable analyses			Multivariable analyses		
	HR	95% CI	*P* value	HR	95% CI	*P* value
CCS/CRF						
Yes versus No	0.56	0.44–0.70	< 0.001	0.57	0.45–0.72	< 0.001
Age (yr)	0.99	0.98–1.00	0.107			
Sex	0.95	0.73–1.23	0.683			
Male versus Female						
BMI						
18.5–23.9 vs < 18.5	0.81	0.57–1.14	0.222			
> = 24 versus < 18.5	0.71	0.49–1.03	0.071			
Primary tumor resection						
Yes versus No	0.93	0.72–1.21	0.600			
Treatment line						
Second line versus First line	1.25	0.95–1.65	0.111	1.17	0.88–1.54	0.278
Third line versus First line	1.71	1.16–2.54	0.007	1.60	1.08–2.37	0.019
ICIs class						
With chemotherapy versus ICIs monotherapy	0.98	0.69–1.40	0.931			
With targeted therapy versus ICIs monotherapy	1.14	0.73–1.79	0.559			
With chemotherapy and targeted therapy versus ICIs monotherapy	0.95	0.60–1.50	0.822			

*CCS* chronic coronary syndromes *CRF* coronary risk factors *BMI* body mass index *ICIs* immune checkpoint inhibitors

### Assessing the safety of ICIs

**Fig. 4:**
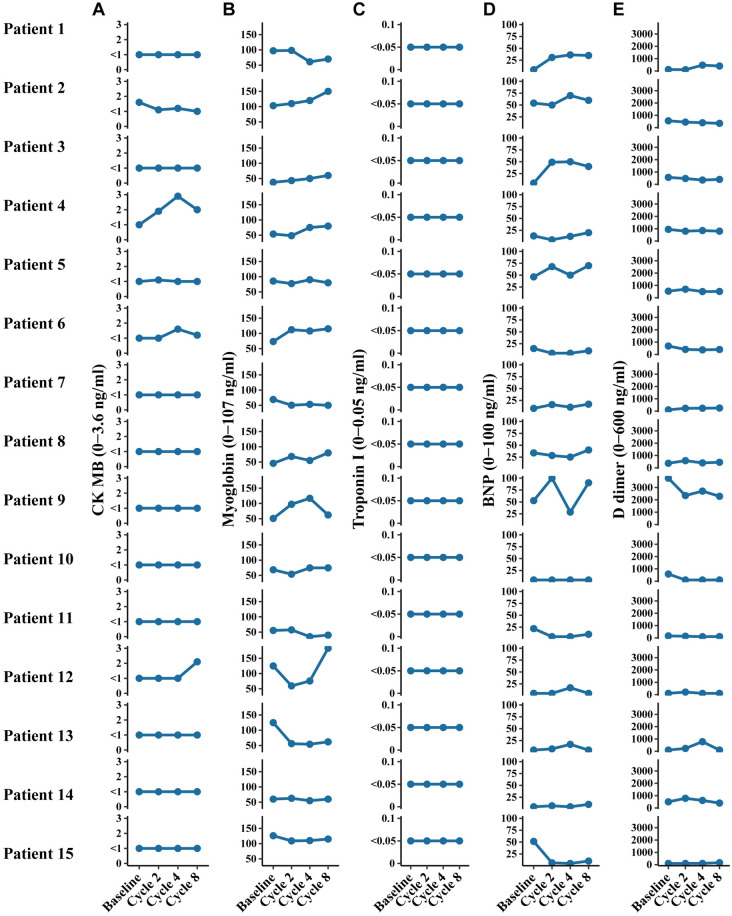
Dynamic changes in cardiac enzyme profiles during treatment in 15 patients with chronic coronary syndrome. The 15 patients included in this study were from the CCS/CRF group at our center (HMUCH). They underwent 8 cycles of ICIs treatment and dynamic cardiac enzyme profile testing. CK-MB, Myoglobin, Troponin I, BNP were determined by automated enzymatic kits on a Dimension EXL 200 Integrated Chemistry System (SIEMENS, Berlin, Germany). D-dimer was measured using an immunoturbidimetric assay (Innovance D-Dimer; Siemens Healthcare) by CN-6000 autoanalyzer (Sysmex, Kobe, Japan).The cardiac enzymes analyzed included: **A** CK-MB (normal range 0–3.6 ng/ml), **B** Myoglobin (normal range 0–107 ng/ml), **C** Troponin I (normal range 0–0.05 ng/ml), **D** BNP (normal range 0–100 ng/ml), and **E** D-dimer (normal range 0–600 ng/ml)

During ICIs therapy, a total of 238 patients (238/551, 43.2%) experienced different level treatment-related adverse events (TRAEs), 103 patients (103/216, 47.7%) of them were from CCS/CRF group and the rest (135/335, 40.3%) were from the non-CCS/CRF group. Only 18 patients (3.3%) occurred with grade ≥ 3 TRAEs and they were successfully managed with symptomatic treatments. The most common abnormal laboratory tests were pruritus/rash (37/551, 6.7%), hypothyroidism (35/551, 6.4%), anemia (34/551, 6.2%). There was no difference in the proportion of TRAEs between the CCS/CRF group with the non-CCS/CRF group (*P* > 0.05), and ST-T changes according to ECG were observed more frequently in the CCS /CRF group than in the non-CCS/CRF group with not exactly significant (5.1% vs. 3.9%, *P* = 0.64). (sTable 4). Additionally, we selected 15 patients from our institution's CCS/CRF group who received 8 cycles of ICIs treatment and underwent dynamic cardiac enzyme profile testing. After ICIs treatment, the changes in serum cardiac markers in patients were shown in Fig. 4A–E. CK-MB, myoglobin, troponin I, BNP and D dimer were not significantly elevated during ICIs treatment, and consistently fluctuated within the normal range.

### Factors affecting the efficacy of ICIs in tumor patients with CCS/CRF

**Fig. 5:**
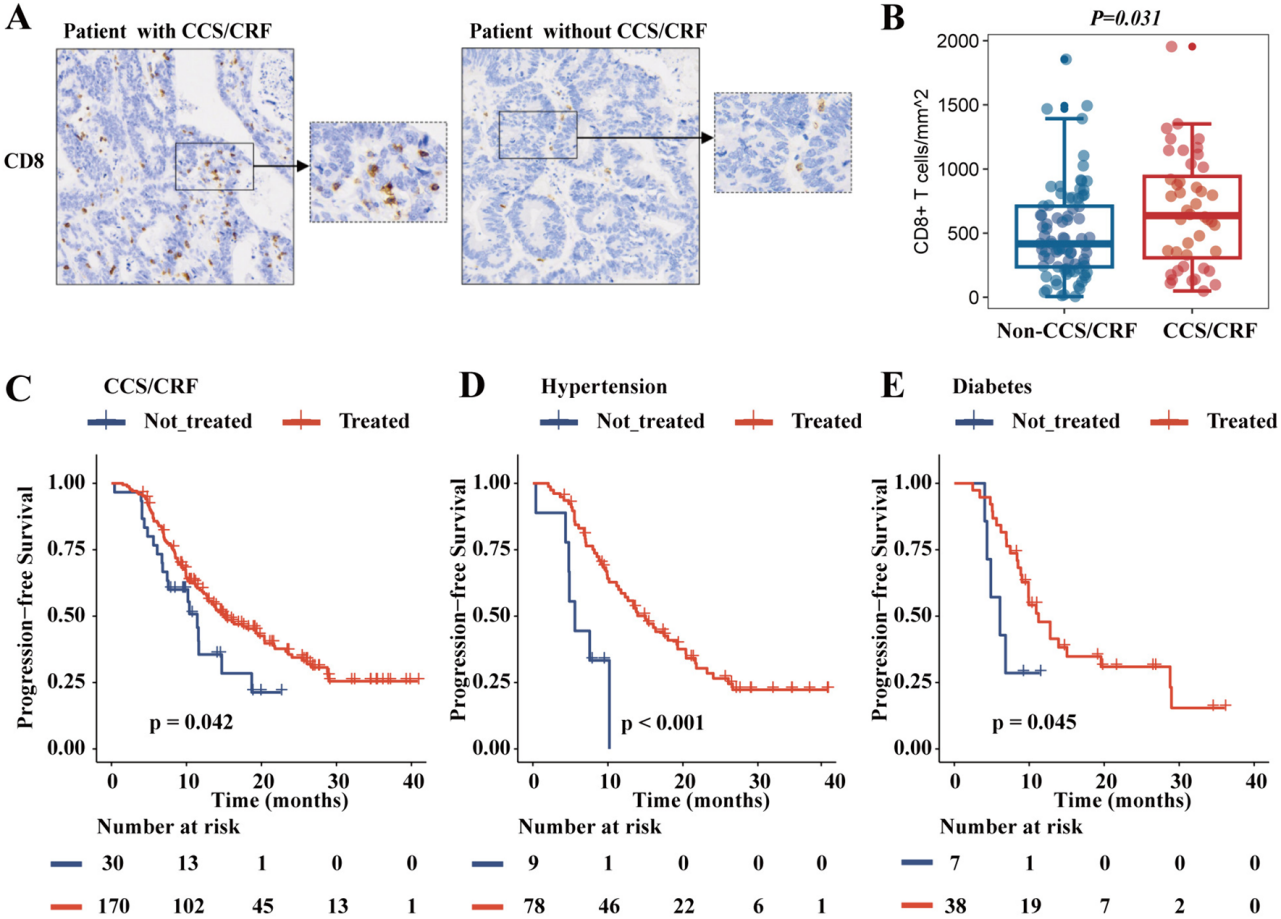
Comparison of the infiltration of immune cells in groups with/without CCS/CRF and survival analysis between treated and untreated groups. Patients included in the study were 125 individuals with stage I-III dMMR CRC from our center (HMUCH). **A** Representative figures of immunohistochemistry for CD8^+^ cells (200×) stained with CD8 antibody of patients with CCS/CRF (left) and without CCS/CRF (right). **B** Comparison of the infiltration of CD8^+^ cell between CCS/CRF group and non-CCS/CRF group. Kaplan–Meier curves of PFS in patients with CCS/CRF **C** hypertension **D** and diabetes **E** a comparative analysis between treatment and untreated groups. CCS: chronic coronary syndromes, CRF: coronary risk factors

To analyze the factors that tumor patients with CCS/CRF benefit from ICIs therapy, we performed IHC and cohort subgroup analysis. We quantified the intratumoral immune infiltrates in 125 pathological tissues of stage I-III dMMR CRC. Characteristics of patients with CRC were detailed in sTable1. Representative immunohistochemistry of CD8 + cells is shown in Fig. 5A. The density of CD8 + lymphocytes was higher in the CCS/CRF group (median, 636, inter-quartile range [IQR]: 307–942) than in the non-CCS/CRF group (median, 417, IQR: 238–711) (*P* = *0.031*) (Fig. 5B). Except for 16 patients for whom medication information could not be obtained, 15% (30/200) of CCS/CRF patients had not receive systematic medical treatment. By comparison, we found that patients in CCS/CRF group who received systematic medical treatment had longer PFS than those who did not (*P* = *0.042*) (Fig. 5C). There were different PFS between hypertension and diabetes patients who received and did not receive medical treatment in CCS/CRF group (*P* < *0.001*, *P* = *0.045*) (Fig. 5D, E).

## Discussion

ICIs block inhibitory T-cell receptors and promote increased anti-tumor activity [23]. These agents, such as CTLA-4, PD-1, and PD-L1 mAb, have transformed cancer treatment and are now approved in over 15 different cancers [24, 25]. However, this increased T-cell activation also contributes to irAEs, which most commonly affect the gut, skin, liver, lungs, and endocrine glands [26]. Incidences of CV-irAEs are rare but fatal. Especially for tumor patients with CCS/CRF, the risk of occurring CV-irAE seems to be more dangerous. Our current study aimed to evaluate the efficacy and safety of ICIs in solid tumors with pre-existing CCS/CRF. We conclude that under the condition that CCS/CRF management is stable, solid tumor patients with pre-existing have a better response to ICIs therapy than those without CCS/CRF. This clinical observation is supported by short-term and long-term survival data. Furthermore, we observed zero cases of CV-irAEs in patients with CCS/CRF during ICIs therapy. In this cohort, we did not observe that CCS/CRF patients had a higher risk of occurring CV-irAEs.

Several studies have confirmed that cardiovascular medicine has other properties for improving the cancer immunological microenvironment and enhancing the anti‐tumor efficacy of ICIs therapy [27–29]. PCSK9 antibody has been approved for human clinical use to lower cholesterol levels [30, 31]. And targeting PCSK9 also increases the expression of major histocompatibility protein class I (MHC-I) proteins on the tumor cell surface, promoting robust intratumoral infiltration of cytotoxic T cells. And PCSK9 antibodies synergize with anti-PD1 therapy in suppressing tumor growth in mouse models of cancer [32]. Metformin is a widely used drug to treat type II diabetes mellitus. It also reduces cancer incidence in patients with diabetes who take the drug [33, 34]. In addition, metformin enhanced the efficacy of immunotherapy by increasing cytotoxic T lymphocyte activity [35]. Another research showed that nifedipine (NIFE), acalcium channel blocker, inhibits calcium influx to impair nuclear factor of activated T cell 2 (NFAT2) dephosphorylation, activation, and nuclear translocation, thus decreasing the expression of PD-L1 on CRC cells and reactivates tumor immune monitoring, which may stimulate or enhance PD-1-based antitumor immunotherapy [28]. A study involving 195 metastatic malignant melanoma patients observed prolonged OS in patients who received immunotherapy (IL-2, anti-PD-1, or anti-CTLA-4) and were taking pan beta blockers experienced [36]. Another small-sample study (N = 55) demonstrated metastatic malignant melanoma patients who have received metformin in combination with ICIs experienced favorable treatment-related outcomes (ORR, DCR, median PFS, and median OS) without reaching significance [37].

To explore the potential reasons why CCS/CRF patients benefit more from ICIs therapy, we performed IHC for CRC tumors undergoing radical resection and showed that CD8^+^ T cells were more frequently infiltrating in patients with CCS/CRF than in patients without CCS/CRF. A strong lymphocytic infiltration has been reported to be associated with favorable survival and efficacy of ICIs in many different tumor types, including digestive system neoplasms (colorectal [38–40], esophagus [41, 42], gastric [43], and pancreatic cancer [44]), melanoma [45], breast [46], and lung cancer [47, 48]. Meanwhile, we compared the lifestyle habits of the two groups. To clarify the role of combined treatment in affecting the efficacy of ICIs, we investigated the disease management information of patients with CCS/CRF. 85.0% (170/200) of patients with CCS/CRF were treated with regular and long-term cardiovascular treatment. The common cardiovascular medicines were calcium channel blockers, angiotensin-converting enzyme inhibitors/angiotensin receptor blockers, insulin, and metformin. We proved that patients who received systematic medical treatment for CCS/CRF had longer PFS. Based on this, we speculate that patients with previous CCS/CRF may have a tumor microenvironment with sensitizes ICIs because of the combination of treatment.

With the advent of ICIs therapy, one of the most historic leaps in medicine in recent years, adverse reactions such as irAEs have been reported [49, 50]. The treatment-related toxicity has less common but dreaded effects on the heart. A systematic review evaluated 99 cases of cardiotoxicity with the use of checkpoint inhibitors and the overall case fatality rate was 35% [51]. However, our study supports the safety of ICIs therapy in patients with solid tumors and pre-existing CCS/CRF. All grade ≥ 3 TRAEs were successfully managed with symptomatic treatments and no patients discontinued treatments due to fatal adverse events. We observed zero cases of CV-irAEs such as myocarditis, pericarditis, and arrhythmias. Evidence suggested that irAEs may represent one such clinical biomarker for ICIs response [8]. Lei Xu et al. [52] conducted a retrospective study that included 74 patients and concluded that developing hypothyroidism during programmed death 1 inhibitors therapy is a predictive factor of superior antitumor efficacy in hepatocellular carcinoma. In our study, only a few patients in CCS/CRF group had anomaly indicators, the association between irAEs and ICIs response for patients with solid tumors and pre-existing CCS/CRF was not analyzed. Prospective, multicenter, large-sample studies were needed to verify the efficacy and safety of ICIs therapy in solid tumor patients with pre-existing CCS/CRF. In summary, based on the real-world study, we concluded that the ICIs appear to have better efficacy in malignant solid tumor patients with pre-existing CCS/CRF and are not accompanied by more serious irAEs. However, further prospective clinical trials are still needed to determine safety.

### Supplementary Information

Below is the link to the electronic supplementary material.

Supplementary file1 (TIF 6102 KB)

Supplementary file2 (DOCX 18 KB)

Supplementary file3 (DOCX 16 KB)

Supplementary file4 (DOCX 20 KB)

## Data Availability

All data supporting the findings in this study are presented in the manuscript and the supplementary information, and additional raw data can be made available by the corresponding author upon reasonable request.
