# Supplementary figures and images for: Efficacy and safety of immune checkpoint inhibitors in solid tumor patients combined with chronic coronary syndromes or its risk factor: a nationwide multicenter cohort study
Source: Cancer Immunol Immunother. 2024 Jun 8;73(8):159. doi: 10.1007/s00262-024-03747-w (PMC11162406; doi:10.1007/s00262-024-03747-w)

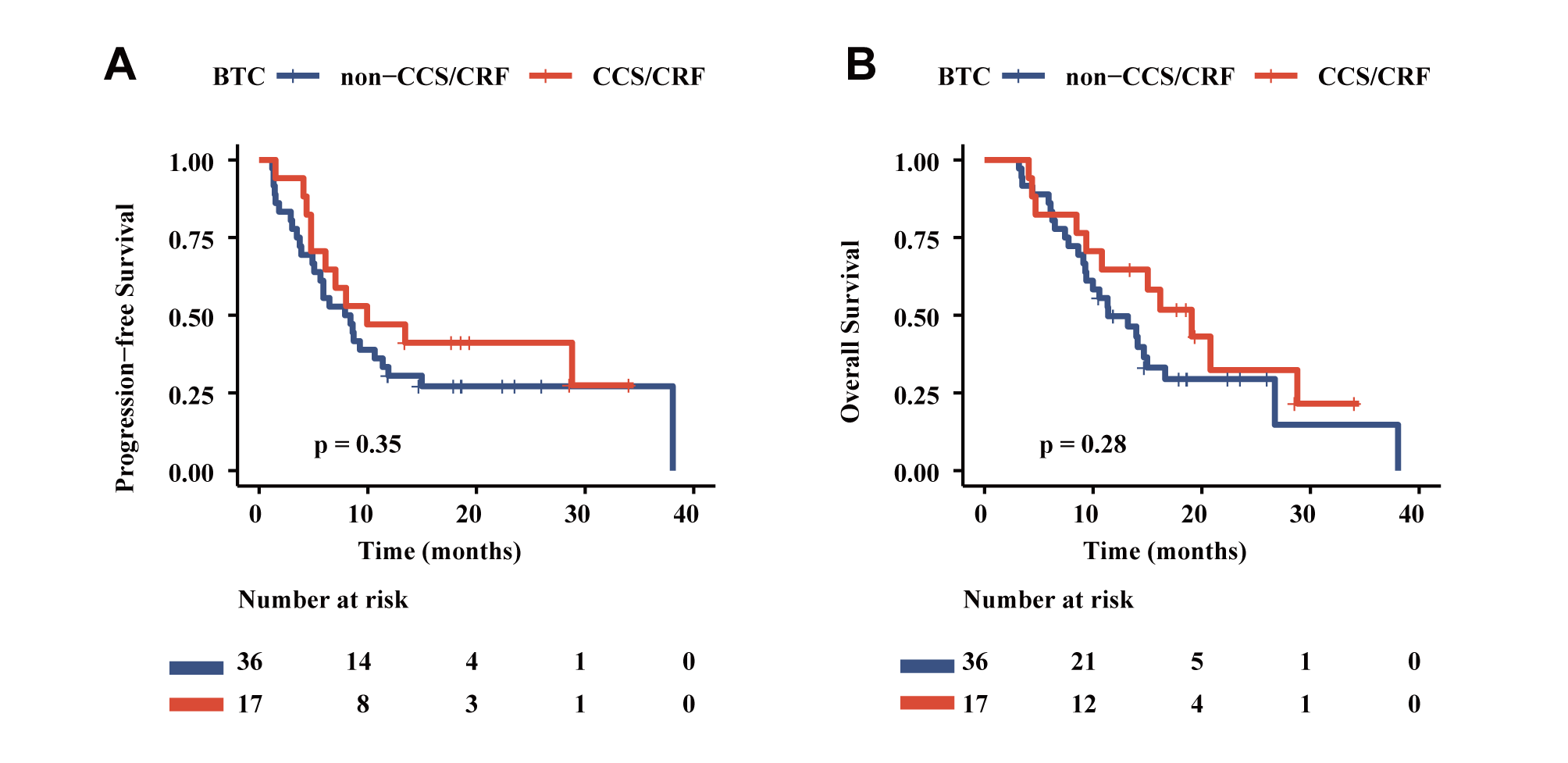

Supplement: Supplementary file 1 — Supplementary file1 (TIF 6102 KB) [file 262_2024_3747_MOESM1_ESM.tif]
