# Supplementary material for: Efficacy and safety of immune checkpoint inhibitors in solid tumor patients combined with chronic coronary syndromes or its risk factor: a nationwide multicenter cohort study
Source: Cancer Immunol Immunother. 2024 Jun 8;73(8):159. doi: 10.1007/s00262-024-03747-w (PMC11162406; doi:10.1007/s00262-024-03747-w)
Supplement: Supplementary file 2 — Supplementary file2 (DOCX 18 KB) [file 262_2024_3747_MOESM2_ESM.docx]

| **Clinicopathological features** | **Non-CCS /CRF(N=81)** | **CCS/CRF (N=44)** | ***P value*** |
| --- | --- | --- | --- |
| Sex |  |  | 0.975 |
| Female | 37(45.7) | 21(47.7) |  |
| Male | 44(54.3) | 23(52.3) |  |
| Age | 50.0[42.0,61.0] | 61.5[56.0,67.0] | <0.001 |
| Location |  |  | 0.974 |
| Right Colon | 51(63.0) | 28(63.6) |  |
| Left Colon | 16(19.8) | 8(18.2) |  |
| Rectum | 14(17.3) | 8(18.2) |  |
| Differentiation |  |  | 0.729 |
| Low | 8(9.88) | 5(11.4) |  |
| Moderate | 64(79.0) | 32(72.7) |  |
| High | 9(11.1) | 7(15.9) |  |
| Neural Invasion |  |  | 0.193 |
| No | 76(93.8) | 38(86.4) |  |
| Yes | 5(6.17) | 6(13.6) |  |
| Vascular Embolism |  |  | 1 |
| No | 77(95.1) | 42(95.5) |  |
| Yes | 4(4.94) | 2(4.55) |  |
| T |  |  | 0.971 |
| 1 | 3(3.70) | 1(2.27) |  |
| 2 | 12(14.8) | 5(11.4) |  |
| 3 | 63(77.8) | 36(81.8) |  |
| 4 | 3(3.70) | 2(4.55) |  |
| N |  |  | 0.317 |
| 0 | 70(86.4) | 34(77.3) |  |
| 1 | 9(11.1) | 7(15.9) |  |
| 2 | 2(2.47) | 3(6.82) |  |
| Stage |  |  | 0.264 |
| I | 14(17.3) | 5(11.4) |  |
| II | 57(70.4) | 29(65.9) |  |
| III | 10(12.3) | 10(22.7) |  |
| CD8+ T cells/mm^2 | 417[238,711] | 636[307,943] | 0.031 |

**Table 1. Clinicopathologic factors of 125 patients with dMMR colorectal cancer undergoing R0 radical surgery at our center, HMUCH.**

CCS: chronic coronary syndromes, CRF: coronary risk factors
