# Supplementary material for: Efficacy and safety of immune checkpoint inhibitors in solid tumor patients combined with chronic coronary syndromes or its risk factor: a nationwide multicenter cohort study
Source: Cancer Immunol Immunother. 2024 Jun 8;73(8):159. doi: 10.1007/s00262-024-03747-w (PMC11162406; doi:10.1007/s00262-024-03747-w)
Supplement: Supplementary file 3 — Supplementary file3 (DOCX 16 KB) [file 262_2024_3747_MOESM3_ESM.docx]

**sTable 2. Cross-Analysis of disease characteristics in CCS/CRF patients from 8 nationwide hospitals with various combinations: a statistical overview of CCS, dyslipidemia, hypertension, and diabetes Mellitus.**

| CCS  (N=28) | Dyslipidemia (N=87) | hypertension (N=93) | Diabetes mellitus (N=47) | Number |
| --- | --- | --- | --- | --- |
| Yes | No | No | No | 12 |
| Yes | No | No | Yes | 1 |
| Yes | No | Yes | No | 2 |
| Yes | No | Yes | Yes | 2 |
| Yes | Yes | No | No | 11 |
| No | No | No | Yes | 26 |
| No | No | Yes | No | 76 |
| No | No | Yes | Yes | 10 |
| No | Yes | No | No | 66 |
| No | Yes | No | Yes | 7 |
| No | Yes | Yes | No | 2 |
| No | Yes | Yes | Yes | 1 |

CCS: chronic coronary syndromes.
