# Supplementary material for: Efficacy and safety of immune checkpoint inhibitors in solid tumor patients combined with chronic coronary syndromes or its risk factor: a nationwide multicenter cohort study
Source: Cancer Immunol Immunother. 2024 Jun 8;73(8):159. doi: 10.1007/s00262-024-03747-w (PMC11162406; doi:10.1007/s00262-024-03747-w)
Supplement: Supplementary file 4 — Supplementary file4 (DOCX 20 KB) [file 262_2024_3747_MOESM4_ESM.docx]

**Table 4. Immune-related adverse events during immune checkpoint inhibitors therapy in 551 patients from 8 nationwide hospitals.**

|  | **Total(N=551)** | **CSS/CRF**  **(N=216)** | **Non_CSS/CRF**  **(N=335)** | ***P value*** |
| --- | --- | --- | --- | --- |
| All TRAEs, N (%) | 238(43.2) | 103(47.7) | 135(40.3) | 0.105 |
| Grade≥3 | 18(3.27) | 11(5.09) | 7(2.09) | 0.091 |
| Blood system disorders |  |  |  |  |
| Anemia, N (%) | 34(6.17) | 10(4.63) | 24(7.16) | 0.305 |
| Thrombocytopenia, N (%) | 10(1.81) | 3(1.39) | 7(2.09) | 0.747 |
| Leukopenia, N (%) | 29(5.26) | 14(6.48) | 15(4.48) | 0.405 |
| Multiple adverse reactions, N (%) | 5(0.91) | 1(0.46) | 4(1.19) | 0.653 |
| Endocrine disorders |  |  |  |  |
| Hyperthyroidism, N (%) | 9(1.63) | 5(2.31) | 4(1.19) | 0.324 |
| Hypothyroidism, N (%) | 35(6.35) | 17(7.87) | 18(5.37) | 0.32 |
| Gastrointestinal disorders |  |  |  |  |
| Diarrhea, N (%) | 12(2.18) | 6(2.78) | 6(1.79) | 0.552 |
| Alanine aminotransferase increased, N (%) | 2(0.36) | 1(0.46) | 1(0.30) | 1 |
| Aspartate aminotransferase increased, N (%) | 18(3.27) | 7(3.24) | 11(3.28) | 1 |
| Multiple adverse reactions, N (%) | 10(1.81) | 5(2.31) | 5(1.49) | 0.524 |
| Renal and urinary disorders |  |  |  |  |
| Creatinine increased, N (%) | 8(1.45) | 3(1.39) | 5(1.49) | 1 |
| Proteinuria, N (%) | 15(2.72) | 7(3.24) | 8(2.39) | 0.740 |
| Skin disorders |  |  |  |  |
| Pruritus/Rash, N (%) | 37(6.72) | 17(7.87) | 20(5.97) | 0.487 |
| RCCEP*, N (%) | 22(3.99) | 11(5.09) | 11(3.28) | 0.403 |
| Multiple adverse reactions, N (%) | 4(0.73) | 1(0.46) | 3(0.90) | 1 |
| New-onset morphological ECG≥2 (interval≥3 days) |  |  |  |  |
| ST-T change, N (%) | 24(4.36) | 11(5.09) | 13(3.88) | 0.641 |
| Negative T-wave, N (%) | 13(2.36) | 4(1.85) | 9(2.69) | 0.732 |
| Right bundle branch block, N (%) | 5(0.91) | 1(0.46) | 4(1.19) | 0.653 |
| Multiple adverse reactions, N (%) | 3(0.54) | 0(0.00) | 3(0.90) | 0.284 |

TRAEs: treatment-related adverse events, CCS: chronic coronary syndromes, CRF: cardiovascular risk factors, RCCEP: reactive cutaneous capillary endothelial proliferation, ECG: electrocardiography, *Common Terminology Criteria for Adverse Events (CTCAE) lacks the grading standard for RCCEP, we referred the classification criteria of skin and subcutaneous tissue disorders.
